# Supplementary material for: Addition of Olive Leaf Extract to a Mixture of Algae and Extra Virgin Olive Oils Decreases Fatty Acid Oxidation and Synergically Attenuates Age-Induced Hypertension, Sarcopenia and Insulin Resistance in Rats
Source: Antioxidants (Basel). 2021 Jul 1;10(7):1066. doi: 10.3390/antiox10071066 (PMC8301163; doi:10.3390/antiox10071066)
Supplement: Supplementary file 1 [file antioxidants-10-01066-s001.zip › antioxidants-1262325-supplementary.pdf]

Addition of olive leaf extract to a mixture of algae and extra virgin olive oils decreases fatty acid oxidation and synergically attenuates age-induced hypertension, sarcopenia and insulin resistance in rats

Daniel González-Hedström [1,2], María de la Fuente-Fernández [1], Teresa Priego [3], Ana Isabel Martín [3], Sara Amor [1], Asunción López-Calderón [3], Antonio Manuel Inarejos-García [2], Ángel Luís García-Villalón [1] and Miriam Granado [1,4,*]

[1] Departamento de Fisiología, Facultad de Medicina, Universidad Autónoma de Madrid, 28029 Madrid, Spain; dgonzalez@pharmactive.eu (D.G.-H.); maria.delafuente@uam.es (M.F.-F.); sara.amor@uam.es (S.A.); angeluis.villalon@uam.es (A.L.G.-V.); miriam.granado@uam.es (M.G.)
[2] Pharmactive Biotech Products S.L. Parque Científico de Madrid. Avenida del Doctor Severo Ochoa, 37 Local 4J, 28049 Alcobendas, Madrid; dgonzalez@pharmactive.eu (D.G.-H.); aminarejos@hotmail.com (A.M.I.-G.).
[3] Departamento de Fisiología, Facultad de Medicina, Universidad Complutense de Madrid, Madrid, Spain; tpriegoc@med.ucm.es (T.P.); anabelmartin@med.ucm.es (A.I.M.); alc@med.ucm.es (A.L.-C.).
[4] CIBER Fisiopatología de la Obesidad y Nutrición. Instituto de Salud Carlos III, 28029 Madrid, Spain; miriam.granado@uam.es (M.G.)
* Correspondence: miriam.granado@uam.es (M.G.); Tel.: +34-914-976-974.

Supplementary information

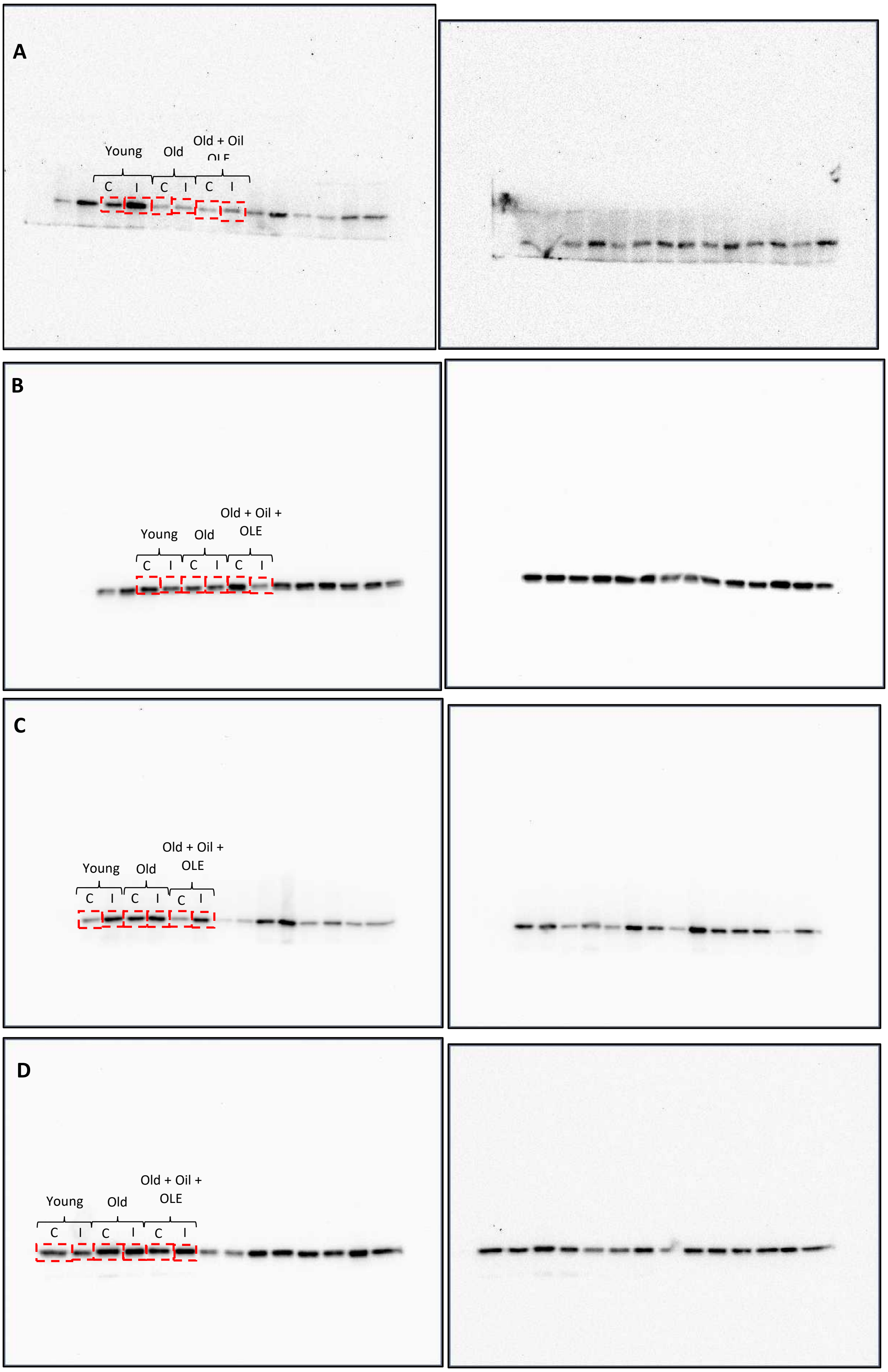

Supplementary Figure 1. Original and unprocessed blots of gastrocnemius p-Akt (**A**) and total Akt (**B**), and epididimal white adipose tissue p-Akt (**C**) and total Akt (**D**) western blots analysis from Figure 3A and B. *Showed blots at Figure 3A and B are marked with red boxes. C = Control; I = Insulin; OLE = olive leaf extract.*
